# High-dimensional causal mediation analysis by partial sum statistic and sample splitting strategy in imaging genetics application

**DOI:** 10.1093/bioinformatics/btaf493

**Published:** 2025-09-10

**Authors:** Hung-Ching Chang, Yusi Fang, Michael T Gorczyca, Kayhan Batmanghelich, George C Tseng

**Affiliations:** Department of Biostatistics, University of Pittsburgh, Pittsburgh, PA 15261, United States; Department of Biostatistics, University of Pittsburgh, Pittsburgh, PA 15261, United States; Department of Biostatistics, University of Pittsburgh, Pittsburgh, PA 15261, United States; Department of Electrical and Computer Engineering, Boston University, Boston, MA 02215, United States; Department of Biostatistics, University of Pittsburgh, Pittsburgh, PA 15261, United States

## Abstract

**Summary:**

Causal mediation analysis investigates the role of mediators in the relationship between exposure and outcome. In the analysis of omics or imaging data, mediators are often high-dimensional, presenting challenges such as multicollinearity and interpretability. Existing methods either compromise interpretability or fail to effectively prioritize mediators. To address these challenges and advance causal mediation analysis in high-dimensional contexts, we propose the Partial Sum Statistic and Sample Splitting Strategy (PS5) framework. Through extensive simulations, we demonstrate that PS5 offers superior type I error control, higher statistical power, reduced bias in mediation effect estimation, and more accurate mediator selection. We apply PS5 to an imaging genetics dataset of chronic obstructive pulmonary disease (COPD) patients from the COPDGene study. The results show successful estimation of the global indirect effect and identification of mediating image regions. Notably, we identify a region in the lower lobe of the lung that exhibits a strong and concordant mediation effect for both genetic and environmental exposures, suggesting potential targets for treatment to mitigate COPD severity caused by genetic and smoking effects.

**Availability and implementation:**

PS5 is publicly available at https://github.com/hung-ching-chang/PS5Med.

## 1 Introduction

Mediation analysis has become an essential tool for uncovering the causal pathways through which exposures affect outcomes. Its importance is particularly evident in biosciences, where high-throughput technologies have enabled the collection of large-scale high-dimensional datasets. These datasets provide profound insights into disease mechanisms, potential biomarkers, and therapeutic targets, ultimately advancing precision medicine. Recent advances in high-dimensional mediation analysis, especially in omics studies (Zeng *et al.* 2021, Clark-Boucher *et al.* 2023, Yang *et al.* 2025), have significantly contributed to the understanding of biological processes. However, few methods have been developed specifically for imaging genetics data, and these mainly focus on neuroimaging (Zhao *et al.* 2020, Chen *et al.* 2022). Our motivating example comes from the chronic obstructive pulmonary disease genetic epidemiology (COPDGene) study (Regan *et al.* 2010), where we aim to investigate whether computed tomography (CT) imaging mediates the impact of a polygenic risk score (PRS) on lung function, measured by forced expiratory volume in one second (FEV1). Unlike most neuroimaging studies, which summarize images into low-dimensional features within pre-specified regions of interest (ROIs), lung imaging lacks predefined functional regions, complicating mediation analysis due to high correlation and dimensionality in the data. As such, there is a pressing need for sophisticated methods that can identify causal pathways while preserving statistical rigor and interpretability in high-dimensional settings.

Current approaches to high-dimensional mediation analysis predominantly rely on dimension reduction or variable selection techniques and can be broadly categorized into two groups: penalized regression (Zhang *et al.* 2016, Zhou *et al.* 2020) and orthogonal transformation (Huang and Pan 2016, Zhao *et al.* 2020). Penalized regression methods reduce mediator dimensionality using sparse priors, which can enhance interpretability. However, these approaches are often limited by their inability to verify causal assumptions or prioritize mediators effectively. For instance, HILMA (Zhou *et al.* 2020) cannot prioritize mediators, and HIMA (Zhang *et al.* 2016) struggles to select highly correlated mediators. In contrast, orthogonal transformation methods transform mediators to be uncorrelated and fit a series of single mediator models to ensure causal assumptions. While these methods maintain statistical rigor, they often sacrifice interpretability and fail to address the challenge of selecting highly correlated mediators, as each transformed mediator is a linear combination of the original mediators. Moreover, orthogonal transformations can lead to a loss of statistical power, particularly when mediators are sparse or highly noisy. In addition to these two categories, recent studies (Dai *et al.* 2022, Liu *et al.* 2022, Tian *et al.* 2022, Roy and Zhang 2024) have focused on improving the false discovery rate (FDR) control and the power of multiple testing procedures, particularly for handling composite null distributions in high-dimensional mediation analysis.

To overcome these challenges and achieve three aims for mediation analysis, we propose a new framework called Partial Sum Statistic and Sample Splitting Strategy (PS5), designed for high-dimensional causal mediation analysis. PS5 addresses four main statistical challenges commonly encountered in the analysis of complex high-dimensional data: (C1) maintaining high statistical power under varying mediation signal structures, (C2) accurately prioritizing and selecting highly correlated mediators, (C3) ensuring causal assumptions are met in feature selection, and (C4) avoiding biased estimation due to collinearity among mediators with high exposure effects. Table 1 outlines shortcomings of existing methods in addressing one or multiple of these challenges, as well as their limitations in achieving the three key aims. To this end, PS5 incorporates partial sum (PS) statistic to improve detection of the global indirect effect (A1), ensuring high statistical power even in sparse mediation scenarios (C1). A sample splitting strategy is combined with penalized regression to ensure preservation of causal assumptions, thus overcoming the challenges related to overfitting and causal assumption violations (C3). Multiple sample splitting further improves mediator selection, effectively identifying highly correlated mediators (C2), which is a typical challenge in omics and imaging data. A residual correction procedure stabilizes mediation effect estimation (A2), successfully reducing the bias in mediator effect estimates (C4). Finally, marginal tests are used to prioritize mediators (A3), facilitating meaningful biological interpretations.

**Table 1.** Methods comparison based on three aims and four challenges.^a^

	A1	A2	A3	C1	C2	C3	C4
Category I: penalized regression							
PS5 (proposed)	✓	✓	✓	✓	✓	✓	✓
Guo2023 (Guo *et al.* 2023)	✗	✓	✓	✗	✗	✗	✓
HIMA2 (Perera *et al.* 2022)	✗	✓	✓	✗	✗	✗	✗
GMM (Song *et al.* 2021)	✗	✓	✓	✗	✗	✓	✓
BSLMM (Song *et al.* 2020)	✗	✓	✓	✗	✗	✓	✓
HIMA (Zhang *et al.* 2016)	✗	✓	✓	✗	✗	✗	✗
PathwayLasso (Zhao and Luo, 2022)	✗	✓	✓	✗	✗	✗	✗
HILMA (Zhou *et al.* 2020)	✓	✓	✗	✗	✗	✓	✓
Category II: orthogonal transformation							
H&P (Huang and Pan 2016)	✓	✗	✗	✗	✗	✓	✗
SPCMA (Zhao *et al.* 2020)	✓	✗	✗	✗	✗	✓	✗
Category III: multiple-testing procedure							
HDMT (Dai *et al.* 2022)	✗	✓	✓	✓	✓	✓	✗
DACT (Liu *et al.* 2022)	✗	✓	✓	✓	✓	✓	✗
MLFDR (Roy and Zhang, 2024)	✗	✓	✓	✓	✓	✓	✗
CoxMKF (Tian *et al.* 2022)	✗	✓	✓	✓	✗	✗	✗

a A1: test for global indirect effect; A2: estimation of global indirect effect; A3: mediators Prioritization; C1: capturing unknown signal structure; C2: feasibility of highly correlated mediators; C3: rigor of causal assumptions; C4: unbiased estimation of mediation contributions.

Through extensive simulations and real-world applications, PS5 demonstrates significant improvements in statistical power, estimation accuracy, and mediator prioritization compared to existing methods. Its application to imaging genetics datasets uncovers novel insights into the causal pathways of complex diseases, such as chronic obstructive pulmonary disease (COPD). By providing a rigorous and interpretable framework for high-dimensional mediation analysis, PS5 addresses critical gaps in the field and offers a powerful tool for researchers to investigate complex biological and clinical questions.

## 2 Materials and methods

**Figure 1.**
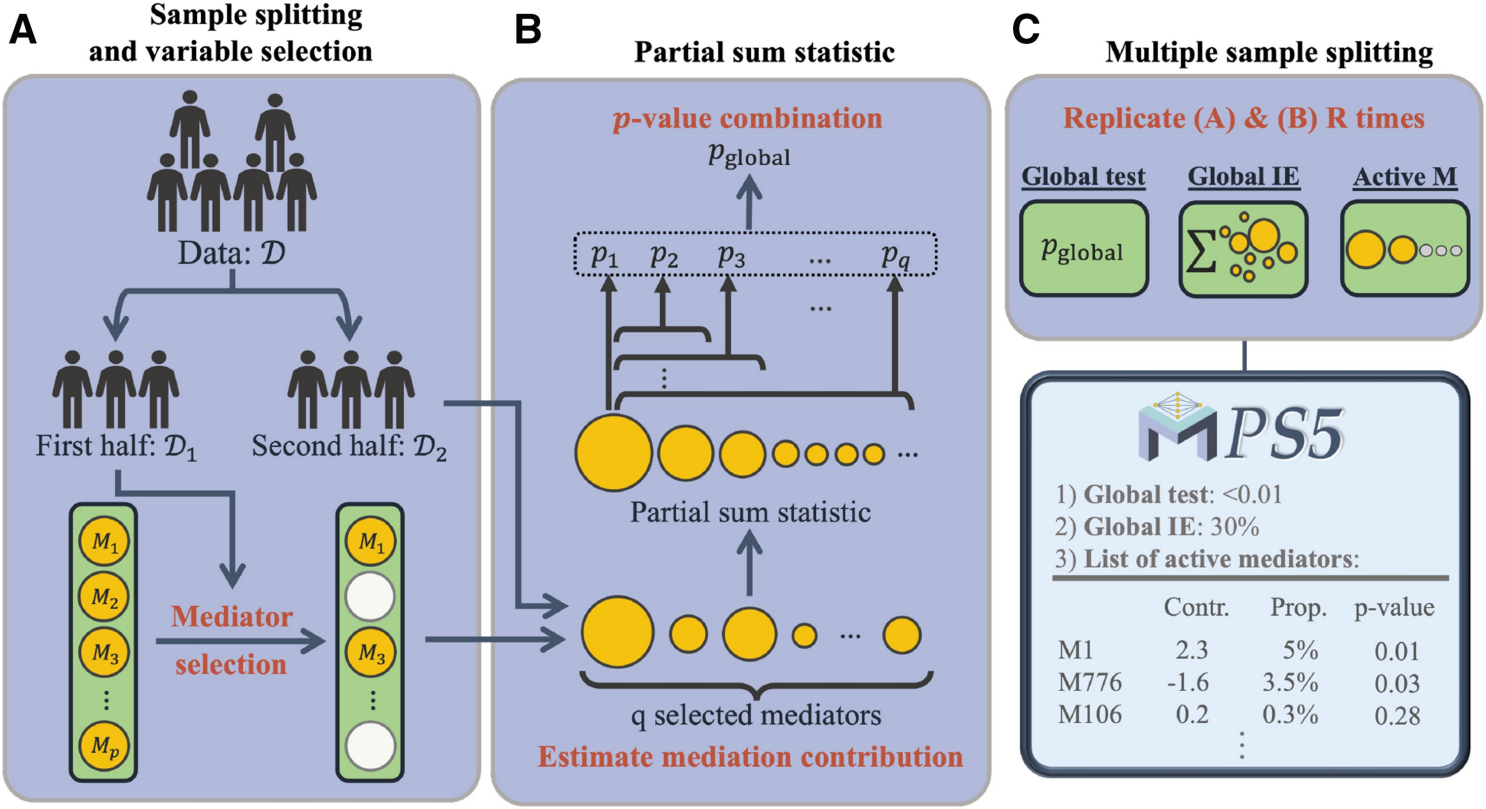
Graphical abstract of PS5, a three-step analysis framework including (A) sample splitting and variable selection, (B) partial sum statistic for testing global indirect effect, and (C) multiple sample splitting and prioritization of selected mediators.

The PS5 framework is built on three major methodological components: sample splitting for variable selection, PS statistics for testing the global indirect effect, and multiple sample splitting for robust mediator prioritization (Fig. 1). The aim of PS5 is to achieve three aims (A1–A3) and to address four challenges (C1–C4) of high-dimensional causal mediation analysis, including sparse signals, high collinearity, and the preservation of causal assumptions. We begin by introducing the notations and causal mediation assumptions that will be used in the rest of the article.

### 2.1 Notations and assumptions

In the context of the high-dimensional mediation model, we collect a dataset of *N* i.i.d. samples, denoted as $D=(X_{1},M_{1},Y_{1},C_{1}),\ldots ,(X_{N},M_{N},Y_{N},C_{N})$. Each sample comprises an exposure variable $X_{i}$, an outcome $Y_{i}$, *l*-dimensional covariates $C_{i}={\left(C_{i}^{\left(1\right)},\ldots ,C_{i}^{\left(l\right)}\right)}^{T}$, and *p*-dimensional mediators $M_{i}={\left(M_{i}^{\left(1\right)},\ldots ,M_{i}^{\left(p\right)}\right)}^{T}$, for i=1,…,N. To define causal mediation effects, we use the (counterfactual) potential outcome framework. Specifically, let $Y_{i}(x,M_{i}(x))$ represent the potential outcome for subject *i* under the exposure level *x*, and $M_{i}(x)={\left(M_{i}^{\left(1\right)}(x),\ldots ,M_{i}^{\left(p\right)}(x)\right)}^{T}$ represent a *p*-dimensional potential mediator for subject *i* given exposure level *x*. The total effect (TE) of exposure *X* on outcome *Y* can be decomposed into the direct effect and the effect mediated through the entire group of mediators, known as the indirect effect. The natural direct effect (NDE) is defined as $Y(x,M(x^{*}))-Y(x^{*},M(x^{*}))$, capturing the effect of *X* on *Y* through pathways that do not involve the mediators **M**. On the other hand, the natural indirect effect (NIE) is defined as $Y(x,M(x))-Y(x,M(x^{*}))$, representing the effect of changing mediators from $M(x^{*})$ to M(x) when exposure is fixed at level *x*. The TE can be decomposed as: $\text{TE}=Y(x,M(x))-Y(x^{*},M(x^{*}))=[Y(x,M(x))-Y(x,M(x^{*}))]+[Y(x,M(x^{*}))-Y(x^{*},M(x^{*}))]=\text{NIE}+\text{NDE}$.

Denote A⊥⊥B|C as *A* independent of *B* conditional on *C*. The sufficient assumptions (Pearl 2001, VanderWeele and Vansteelandt 2014) for identifying causal effects in mediation analysis are: (I) Y(x)⊥⊥X|C, (II) Y(x,m)⊥⊥M|X,C, (III) M(x)⊥⊥X|C, and (IV) $Y(x,m)⊥⊥M(x^{*})|C$. Assumptions (I)–(III) are the no-unmeasured confounding assumptions, while assumption (IV) is known as the cross-world assumption (Andrews and Didelez 2021). Under these assumptions, the average NDE and NIE can be identified through the following regression models for *Y* and M using observed data:


(1) $$Y_{i}=C_{i}^{T}\beta_{C}+X_{i}\beta_{X}+M_{i}^{T}\beta_{M}+\epsilon_{Y_{i}}$$

(2) $$M_{i}=\alpha_{C}C_{i}+X_{i}\alpha_{X}+\epsilon_{M_{i}},$$

where $\epsilon_{Y_{i}}∼N(0,\sigma^{2})$, $\epsilon_{M_{i}}={\left(\epsilon_{M1i},\ldots ,\epsilon_{\mathrm{Mpi}}\right)}^{T}∼N_{p}(0,\Sigma_{M})$, $\beta_{C}{\;}^{T}=(\beta_{C1},\ldots ,\beta_{Cl})$, $\beta_{M}{\;}^{T}=(\beta_{M1},\ldots ,\beta_{Mp})$, $\alpha_{C}={\left(\alpha_{C1},\ldots ,\alpha_{Cp}\right)}^{T}$ a p×l matrix, $\alpha_{X}={\left(\alpha_{X1},\ldots ,\alpha_{Xp}\right)}^{T}$, and $\epsilon_{Y_{i}}$ is assumed to be independent with $\epsilon_{M_{i}}$. Here, we assume there is no interaction between *X* and *M*. Then, NDE and NIE can be expressed as below:


$$\begin{array}{c} E[\text{NDE}]=E[Y(x,M(x^{*}))-Y(x^{*},M(x^{*}))|C]=\beta_{X}(x-x^{*})\\ E[\text{NIE}]=E[Y(x,M(x))-Y(x,M(x^{*}))|C]=\alpha_{X}{\;}^{T}\beta_{M}(x-x^{*}) \end{array}$$

The NDE simply corresponds to the coefficient $\beta_{X}$ in the outcome model (1), and the NIE can be expressed as the sum of the products $\alpha_{Xj}$ and $\beta_{Mj}$, j=1,…,p. For simplicity, we refer to the NDE and NIE as the direct effect and global indirect effect, respectively. To have a more straightforward interpretation, we also define the $\text{GM}\%=\frac{\text{NIE}}{\text{TE}}$ as the global mediation percentage to represent the proportion of the TE explained by the mediators.

### 2.2 PS5: the proposed three-step framework

The previous section described the notations and assumptions for causal mediation model. Below, we provide the details of PS5 for testing, estimating, and prioritizing the mediation effect to achieve (A1) conduct a powerful statistical test to detect the global indirect effect, (A2) quantify the global indirect effect, and (A3) prioritize and select active mediators.

#### 2.2.1 Step 1: sample splitting for mediator selection

To address the challenges of variable selection and ensure valid statistical inference in high-dimensional causal mediation analysis, the sample splitting strategy is a foundational step in the PS5 framework. This approach minimizes the risk of overfitting and overly optimistic *p*-value, which are common in high-dimensional settings (p≫N). The process begins by dividing the dataset into two equal parts: $D_{1}$ and $D_{2}$, each containing N/2 observations. The first subset, $D_{1}$, is used for variable selection to reduce the dimensionality of mediators to a manageable size. The second subset, $D_{2}$, is then used exclusively for estimation and hypothesis testing. This division ensures that the selection process does not bias subsequent inference, a crucial step in maintaining the rigor of the analysis.

One of the main challenges in this step is the high correlation among mediators. This collinearity often arises from substantial exposure effects ($\alpha_{X}$) on mediators, making it difficult to accurately estimate the mediator-outcome relationships ($\beta_{M}$). To mitigate this issue, marginal exposure effects on both the outcome (*Y*) and the mediators (M) are removed prior to variable selection. Specifically, the following regressions are fitted: $Y_{i}=X_{i}\beta_{X}+\epsilon_{1}$ and $M_{i}=X_{i}\alpha_{X}+\epsilon_{2}$. The residuals from these regressions, $\mu_{Y_{i}}=Y_{i}-X_{i}{\hat{\beta }}_{X}$ and $\mu_{M_{i}}=M_{i}-X_{i}{\hat{\alpha }}_{X}$, are then used in subsequent analysis. By isolating the mediator–outcome relationships, this adjustment enhances the stability and accuracy of the estimates, particularly in the presence of strong exposure effects.

After sample splitting and removing the marginal exposure effect, we apply the minimax concave penalty (MCP) method (Zhang 2010) to $D_{1}$. MCP is a regularization technique well-suited for high-dimensional data due to its ability to provide less biased estimates and its theoretical consistency in variable selection. The optimization problem solved by MCP is as follows:


(3) $${\hat{\beta }}_{M}(\lambda )=\underset{\beta_{M}}{\text{arg min}}\sum_{i\in D_{1}}{\left(\mu_{Y_{i}}-C_{i}^{T}\beta_{C}-\mu_{M_{i}}^{T}\beta_{M}\right)}^{2}+\sum_{j}P(\beta_{Mj},\lambda )$$

where $P(\beta_{Mj},\lambda )$ is the regularization penalty. Suppose *q* mediators (denoted as $M^{\prime }\subset M$, q≪p) is selected from $D_{1}$ by MCP. This subset of mediators ($M^{\prime }$) in $D_{2}$ will be used in the following steps. A critical aspect of this approach is the preservation of causal assumptions during variable selection. Proposition 1 guarantees that removing mediators without a mediator–outcome relationship through the MCP procedure does not violate causal assumptions. This theoretical foundation is crucial for ensuring the integrity of the mediation analysis, and the detailed proof is provided in the Supplementary Materials 1, available as supplementary data at *Bioinformatics* online.

Proposition 1. *Given that casual assumptions (I)–(IV) are held for mediators model* $X-(M^{\left(1\right)},\ldots ,M^{\left(p\right)})-Y$*, removing candidate mediators without mediator-outcome relationship (as in the MCP procedure in Equation (3)) can preserve the causal assumptions (I)–(IV).*

#### 2.2.2 Step 2: partial sum statistic for testing global indirect effect

The second step of PS5 focuses on testing the global indirect effect. The null hypothesis of no mediation is defined as:


(4) $$H_{0}:\sum_{j=1}^{q}\alpha_{Xj}\beta_{Mj}=0\;\text{vs}.\;H_{A}:\sum_{j=1}^{q}\alpha_{Xj}\beta_{Mj}\neq 0,$$

where *q* is the number of mediators selected in the previous step and $\alpha_{Xj}\beta_{Mj}$ is also known as the mediation contribution of the *j*th mediator. A direct summation of mediation contributions can suffer power loss due to cancelation of positive and negative effects among mediators. Consequently, we use sum of the Lγ norm of $\alpha_{Xj}\beta_{Mj}$ for the hypothesis test:


(5) $$H_{0}:\sum_{j=1}^{q}|\alpha_{Xj}\beta_{Mj}{|}^{\gamma }=0\;\text{vs}\;.\;H_{A}:\sum_{j=1}^{q}|\alpha_{Xj}\beta_{Mj}{|}^{\gamma }\neq 0$$

We note that the signals detected by $H_{A}$ of Equation (4) are a subset of the $H_{A}$ by Equation (5). For example, a cancelation of effects can happen when the total positive effects of $\alpha_{Xj}\beta_{Mj}$ equals the total negative effects, which results in $H_{0}$ in Equation (4) but $H_{A}$ in Equation (5). To better quantify cancelation effects, we introduce a measure of the neutralization ratio (NR) defined as $\text{NR}=1-\frac{\text{IE}}{\left|{\text{IE}}^{+}|+|{\text{IE}}^{-}\right|}$, where $\text{IE}=\sum_{j=1}^{q}\alpha_{Xj}\beta_{Mj}$ represents the global indirect effect, ${\text{IE}}^{+}=\sum_{j=1}^{q}\alpha_{Xj}\beta_{Mj}I(\alpha_{Xj}\beta_{Mj}\geq 0)$, and ${\text{IE}}^{-}=\sum_{j=1}^{q}\alpha_{Xj}\beta_{Mj}I(\alpha_{Xj}\beta_{Mj}<0)$. Table 1, available as supplementary data at *Bioinformatics* online, provides illustrative examples of NR to clarify its computation and biological interpretation.

To improve statistical power, below we design the PS statistic for the hypothesis test in Equation (5). PS statistic orders mediators by the absolute magnitude of their contributions and aggregates them using a Lγ norm to reduce the impact of noise:


$$PS_{k}=\sum_{j=1}^{k}{\left(T_{\left(j\right)}\right)}^{\gamma },$$

where $T_{j}=|\alpha_{Xj}\beta_{Mj}|$, $T_{\left(j\right)}$ is the order statistic of $T_{j}$, γ is a parameter to emphasize stronger signals, and k=1,…,q. The *p*-value for PS statistic $PS_{k}$, denoted as $p_{k}$, can be computed through a Monte Carlo simulation under the null hypothesis. The global mediation test then combines the *p*-values from all PS statistics ($PS_{1},\ldots ,PS_{q}$) using a Cauchy combination test: $T_{PS}(\vec{p})=\frac{1}{q}\sum_{k=1}^{q}\;\text{tan}\;((0.5-p_{k})\pi )\overset{H_{0}}{∼}\text{Cauchy}(0,1)$. The *p*-value from the global mediation test is then calculated as $p_{\text{global}}=1-F_{\text{Cauchy}(0,1)}^{-1}(T_{PS}(\vec{p}))$. We note that a natural choice for the final combination method could simply by taking the minimum: $T_{PS}^{\min}(\vec{p})=\min_{k}p_{k}$ (Li and Tseng 2011). But since $p_{k}$’s are dependent, its null distribution has no closed form and requires a second layer of Monte Carlo simulation, making it computationally infeasible in practice. In contrast, the Cauchy combination method has been shown a robust method for combining dependent, sparse, and weak signals (Fang *et al.* 2024) with null distribution still being a Cauchy distribution. This method is sensitive and robust to detect global signal if any *p*-value in $\vec{p}$ is small.

To calculate $p_{k}$, we adopt a Monte Carlo method similar to Huang and Pan (2016). Denote ${\hat{\alpha }}_{X}$ and ${\hat{\beta }}_{M}$ as the maximum likelihood estimator (MLE) of $\alpha_{X}$ and $\beta_{M}$ under the parametric models (1) and (2) using the original data D and the second half data $D_{2}$. Firstly, approximate the joint distribution of ${\hat{\alpha }}_{X}$ and ${\hat{\beta }}_{M}$ by a multivariate normal distribution:


$$\begin{array}{c} \left(\begin{array}{c} {\hat{\alpha }}_{X}\\ {\hat{\beta }}_{M} \end{array}\right)∼N_{2q}\left(\left(\begin{array}{c} {\hat{\alpha }}_{X}\\ {\hat{\beta }}_{M} \end{array}\right),\left(\begin{array}{cc} \hat{\mathrm{Cov}}({\hat{\alpha }}_{X}) & 0\\ 0 & \hat{\mathrm{Cov}}({\hat{\beta }}_{M}) \end{array}\right)\right), \end{array}$$

given that $\epsilon_{Y}$ and $\epsilon_{M}$ are independent. Secondly, generate Monte Carlo samples $\alpha_{X}{\;}^{\left(b\right)}$, $\beta_{M}{\;}^{\left(b\right)}$, and centered $T_{j}^{\left(b\right)}(0)=|\alpha_{Xj}^{\left(b\right)}\beta_{Mj}^{\left(b\right)}-\frac{1}{B}\sum_{b}\left\{\alpha_{Xj}^{\left(b\right)}\beta_{Mj}^{\left(b\right)}\right\}|$, where b=1,…,B. Thirdly, calculate the PS statistic for each Monte Carlo sample as $PS_{k}^{\left(b\right)}=\sum_{j=1}^{k}[T_{\left(j\right)}^{\left(b\right)}(0){]}^{\gamma }$, where $T_{\left(j\right)}^{\left(b\right)}(0)$ is the order statistic of $T_{j}^{\left(b\right)}(0)$. Finally, the *p*-value for $PS_{k}$ is calculated as $p_{k}=\frac{1}{B}\sum_{b}1(PS_{k}>PS_{k}^{\left(b\right)})$, which corresponds to the proportion of Monte Carlo samples where the PS statistic is greater than or equal to the observed value.

#### 2.2.3 Step 3: multiple sample splitting and prioritization of selected mediators

To address the variability introduced by random sample splitting and select highly correlated mediators, PS5 employs multiple iterations of the sample splitting process proposed by Meinshausen *et al.* (2009). In high-dimensional mediator settings, only one or a few highly correlated active mediators can be selected in each random sample splitting. To address this, we perform multiple sample splitting by running the sample splitting process *R* times in parallel. Combining results from different $D_{1}$ in each split allows us to capture highly correlated and true mediators. Furthermore, to reduce estimation bias from single sample splitting, we take the median of the estimated global indirect effect from multiple iterations. This process helps us achieve more robust and stable results, particularly in cases with highly correlated mediators, by ensuring that our inference is not influenced by the specific data partitioning. In our experience, R=50 is sufficient to generate a stable result while limiting the computational burden.

Based on the mediator sparsity assumption, only a few active mediators contribute to the global indirect effect. Prioritizing these key mediators is critical for biological interpretation and decision-making. Clark-Boucher *et al.* (2023) recently pointed out that $\alpha_{Xj}\beta_{Mj}$ cannot be directly interpreted as a “causal effect” through the *j*th mediator. Instead, $\alpha_{Xj}\beta_{Mj}$ is named as “mediation contribution” and reflects the active mediation level of the *j*th mediator, which will be the basis for our prioritization. Following the estimation and inference procedure described in Step 2, we can calculate the marginal *p*-value of each mediation contribution $\alpha_{Xj}\beta_{Mj}$ using Monte Carlo method: $p_{M_{j}}=\frac{1}{B}\sum_{b}1(|T_{j}|>|T_{j}^{\left(b\right)}|)$.

To avoid the “*p*-value lottery”, we use the *p*-value aggregation method, an empirical δ-quantile method suggested by Dezeure *et al.* (2015), to integrate multiple sample splitting results: $p_{\text{global},\text{agg}}=\text{min}\{Q(0.5,p_{\text{global}}),1\}$, and $p_{M_{j},\text{agg}}=\text{min}\{Q(\delta ,p_{M_{j}}),1\}$, where Q(δ,p) is the empirical δ-quantile of *p*-value vector from *R* multiple sample splitting, with δ set as half the selected proportion. For example, if mediator $M^{\left(1\right)}$ is selected *h* times over *R* multiple sample splitting, δ would be set as 0.5h/R. The FDR and family-wise error rate (FWER) are further controlled by the Benjamini–Yekutieli and Bonferroni procedures, respectively.

## 3 Simulation studies

To evaluate the performance of the proposed PS5 framework, we conducted a series of simulation studies comparing it with three popular methods reviewed in previous studies (Zeng *et al.* 2021, Clark-Boucher *et al.* 2023), namely H&P (Huang and Pan 2016), HIMA (Zhang *et al.* 2016), and HILMA (Zhou *et al.* 2020). The simulation studies were designed under a moderate sample size (*N *= 500) and high-dimensional mediators (*P *= 1000) to assess type I error control, statistical power, bias in estimating the global indirect effect, and sensitivity for mediator prioritization. These simulations provide a comprehensive evaluation of PS5 under various conditions, including different levels of sparsity, signal strengths, and mediator correlation structures.

To mimic PRS exposure, we sample the exposure *X* from N(0,1). The error term $\epsilon_{Y}$ is generated from N(0,1), and the error terms $\epsilon_{M}={\left(\epsilon_{M1},\ldots ,\epsilon_{Mp}\right)}^{T}$ are generated $\mathrm{MVN}(0,\Sigma_{M})$, where $\Sigma_{M}{\;}_{\left(a,b\right)}={\left(\rho^{\left|a-b\right|}\right)}_{a,b}$ with ρ=0 or 0.5. We then generate *p* mediators M and outcome *Y* by using models (1) and (2) with different $\alpha_{X}$ and $\beta_{M}$. We consider the simulation setting in Dai *et al.* (2022) to evaluate type I error under the complete nulls, dense nulls, sparse nulls, and disjunctive nulls:

(Complete nulls): $\alpha_{X}=\beta_{M}=0$

(Dense nulls): $\alpha_{X}∼U(1,3);\beta_{M}=0$

(Sparse nulls): $\alpha_{X}=0;\beta_{M1},\ldots ,\beta_{M50}∼U(1,3);$


$$\beta_{M51},\ldots ,\beta_{M1000}=0$$

(Disjunctive nulls): $\alpha_{X1},\ldots ,\alpha_{X50}=0;$


$$\alpha_{X51},\ldots ,\alpha_{X1000}∼U(1,3);$$

$$\beta_{M1},\ldots ,\beta_{M50}∼U(1,3);$$

$$\beta_{M51},\ldots ,\beta_{M1000}=0$$

**Figure 2.**
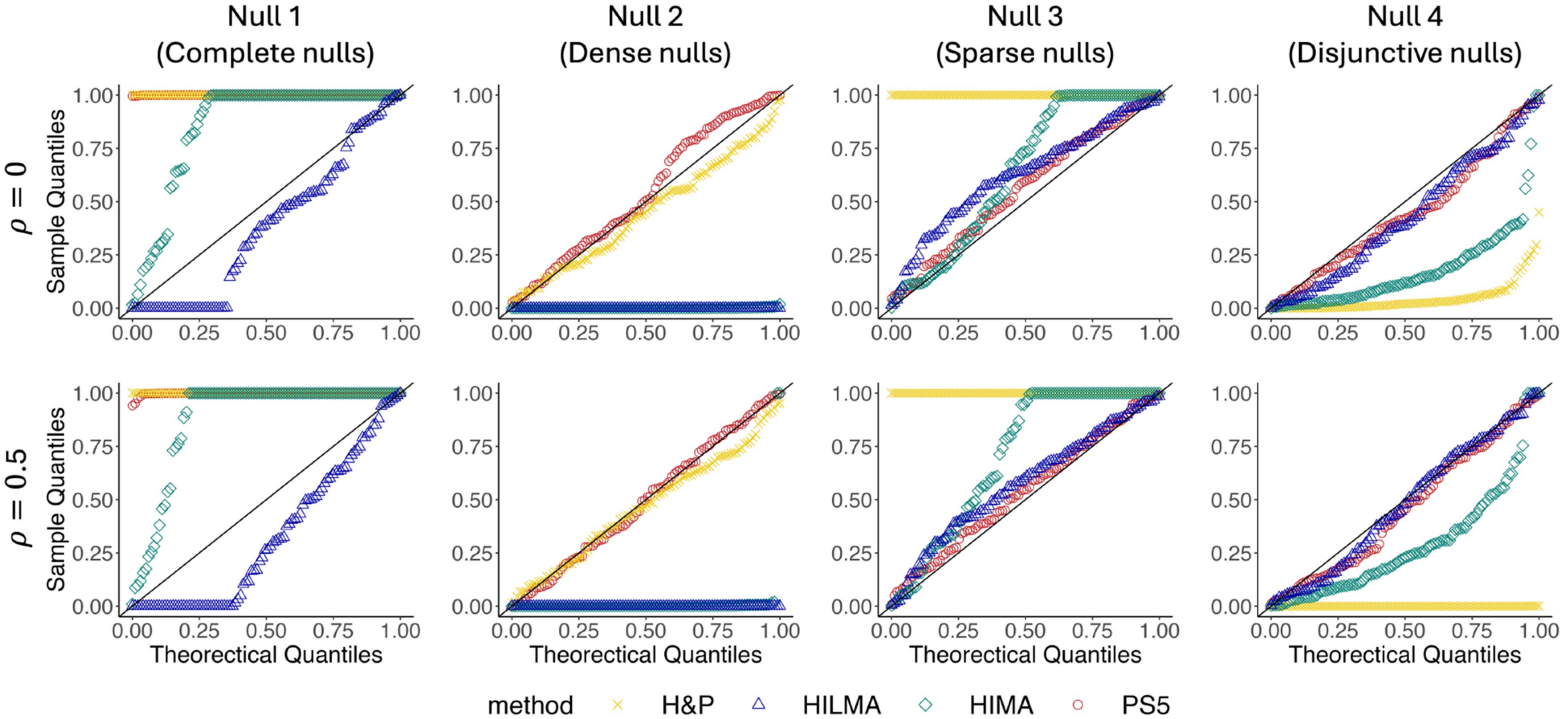
*Q*–*Q* plots of *P*-values under four null cases and two correlation settings (ρ=0,0.5).

Due to the computational burden of HILMA in some null cases, we only replicate 100 simulations for HILMA, whereas other methods are replicated 2000 times. The *Q*–*Q* plots in Fig. 2 and Table 2, available as supplementary data at *Bioinformatics* online, present type I error results. According to the *Q*–*Q* plots, all methods are either too conservative or overly anti-conservative under complete nulls due to the composite null hypothesis, which remains a challenge in high-dimensional mediation testing. While PS5 demonstrated consistent type I error control across all the other null cases, other methods exhibited limitations. For instance, H&P is conservative under sparse nulls but anti-conservative under disjunctive nulls, as it treats disjunctive effects as true mediation effects. HIMA is anti-conservative under dense and disjunctive nulls because it uses the same dataset for dimension reduction and inference, which is a typical overoptimism problem in high-dimensional inference (Meinshausen *et al.* 2009). HILMA is severely anti-conservative when none of the $\beta_{M}$ exist, such as in complete and dense nulls.

**Figure 3.**
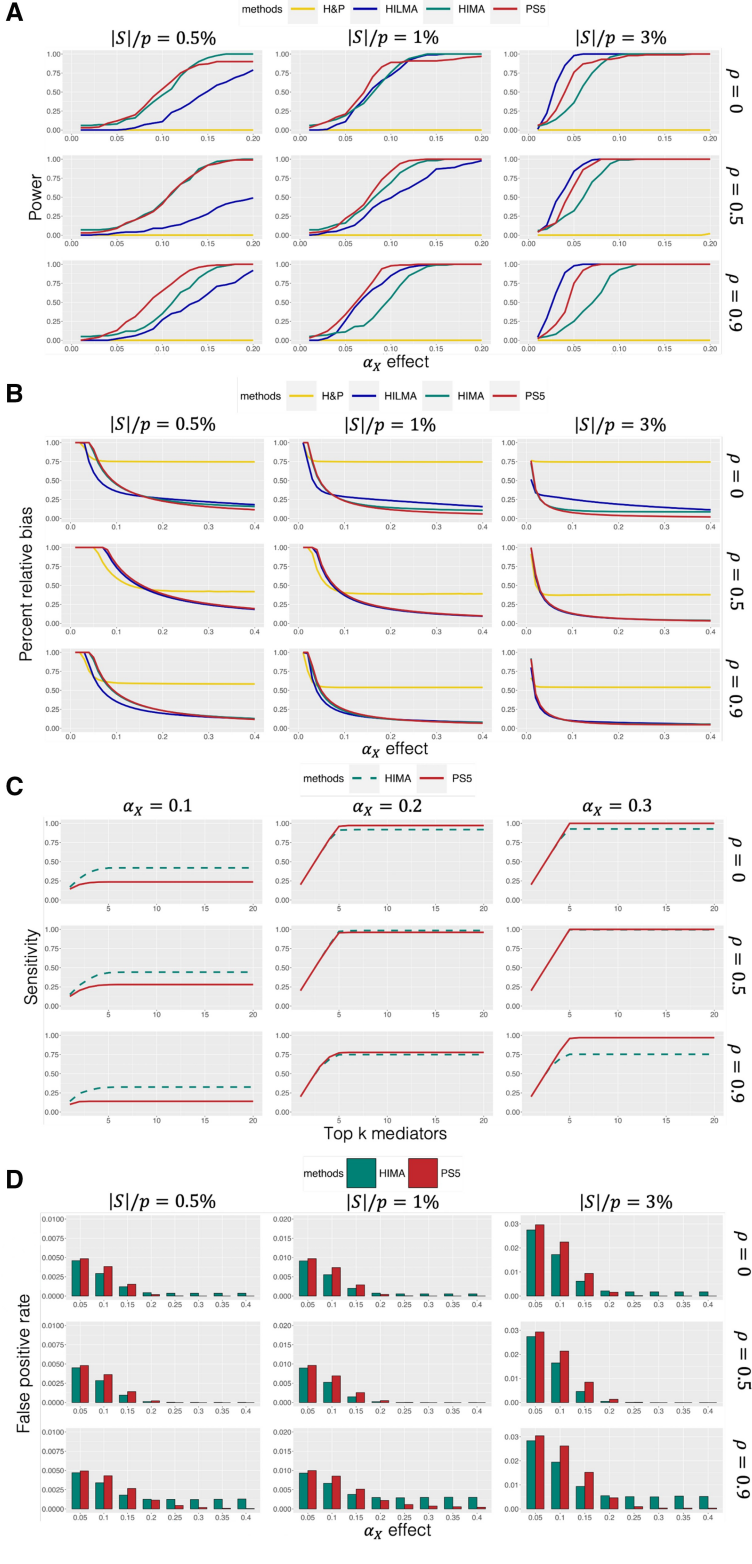
(A) Power for detecting global indirect effect. (B) Percent relative bias for estimating global indirect effect. (C) Sensitivity for mediator prioritization under |S|/p=0.5%. (D) False positive rate for mediator selection.

To assess power, we design alternative hypotheses with various sparsity levels, signal strengths, and correlation structures. In each scenario, the first 50 mediators have $\beta_{M}$ effect ($\beta_{M1}=\ldots =\beta_{M50}=1$), while a subset S has $\alpha_{X}$ effect (i.e. S=1,…,s, where s≤50). Signal strengths for $\alpha_{X}$ range from 0 to 0.2, and three correlation structures are considered: two AR1 models (ρ=0 or 0.5) and one block correlation matrix. The block correlation is designed to simulate the true mediators with high correlation by grouping them into pairs with strong correlation (ρ=0.9). Figure 3A shows the power results under three correlation settings (ρ=0,0.5, 0.9) and three signal structures (|S|/p=0.5%, 1%, 3%). In sparse scenarios (|S|/p=0.5%), HIMA and PS5 are more powerful than HILMA and H&P. However, the power of HIMA decreases as correlation increases. When the number of true mediators |S| increases, the power of HIMA becomes lower than PS5 and HILMA. Overall, PS5 is consistently among the most powerful methods across all scenarios. Notably, the power of PS5 increases with mediator–mediator correlation (ρ). This trend can be explained by two factors: First, PS5’s feature selection step mitigates multicollinearity by retaining one or a few representative mediators from correlated mediators. Second, correlated mediators in our simulations share the same direction of mediation effect, allowing a selected mediator to “borrow” signal from its correlated, non-selected neighbors.

The accuracy of estimating the global indirect effect (IE) is evaluated by computing the relative bias: $\frac{\left|\hat{\text{IE}}-\text{IE}\right|}{\text{IE}}$. Simulations were conducted with increasing $\alpha_{X}$ values and varying correlation and sparsity levels. Figure 3B shows that PS5 demonstrated low bias across all settings, comparable to HIMA and HILMA. In contrast, H&P consistently shows higher bias regardless of the correlation.

The sensitivity of mediator prioritization was assessed by evaluating the proportion of true mediators correctly identified among the top *k* mediators. Since HILMA and H&P do not provide *p*-values for individual mediators, the comparison was limited to PS5 and HIMA. While HIMA achieved slightly higher sensitivity under weak signals, PS5 outperformed as signal strength increased (Fig. 3C). Notably, PS5 reached 100% sensitivity in scenarios with strong correlations, where HIMA struggled to select all true mediators due to its penalized regression approach. In Fig. 3D, we further evaluated the false positive rate (FPR), and again PS5 demonstrated lower FPR than HIMA as signal strength increased, particularly in scenarios with strong correlation. PS5’s success in detecting correlated mediators is attributed to its multiple sample splitting strategy, which aggregates results across splits to capture all active mediators.

We also perform additional simulations in the Supplementary Materials, available as supplementary data at *Bioinformatics* online, including comparisons of the γ parameter, sensitivity analysis with an unmeasured confounding variable, and simulations for discrete exposure. First, the simulation of the γ parameter shows that a larger γ is more powerful for detecting sparse signals. Second, HILMA is the most robust method for handling unmeasured confounders, but PS5 is comparable to HILMA as the number of true mediators |S| increases. Lastly, similar to the continuous exposure setting, PS5 is more powerful and has lower estimation bias in the discrete exposure setting.

## 4 Application to COPDGene study

COPD is the third leading cause of mortality worldwide, accounting for 3 million deaths in 2019 alone (Mei *et al.* 2022). While environmental and social factors, such as cigarette smoking, are widely recognized as major contributors to COPD susceptibility (Salvi 2014), the disease’s heterogeneity has increased interest in identifying its genetic underpinnings. Genome-wide association studies (GWAS) have identified numerous single nucleotide polymorphisms (SNPs) associated with COPD risk, though the effect size of each SNP is typically small (Pillai *et al.* 2009, Cho *et al.* 2014). To better understand the genetic and environmental contributions to COPD, the COPDGene consortium (Regan *et al.* 2010) provides a large dataset comprising comprehensive genetics and CT imaging data, offering a unique opportunity to study the mediating role of lung imaging features.

In this study, we analysed data from COPDGene (*N* = 8897), focusing on the mediating role of CT imaging in the relationship between COPD-related exposures and disease severity, as measured by forced expiratory volume in one second (FEV1). The CT imaging data is first pre-processed by a self-supervised representation learning method (Li *et al.* 2021), which generates 128 representations for each of 581 patches (local regions), resulting in 128 × 581 = 74 368 features. Principal component analysis (PCA) is then applied to reduce the dimensionality of these representations, retaining the first 10 principal components from each patch, which explain approximately 80% of the variance. This process yielded 5810 candidate mediators, with each principal component labeled by its patch index. For example, “M90-1” refers to the first principal component of the 90th patch. Two exposures are analysed in separate mediation models: (1) a polygenic risk score (PRS) derived from GWAS (Moll *et al.* 2020) representing genetic susceptibility and (2) cigarette smoke exposure measured in pack-years (PY), widely regarded as the most important causative factor. Both models include covariates commonly used in COPD research, such as sex, age, and height, along with the top five principal components from genotype data (SNPs) to adjust for population stratification. Importantly, PRS and PY were uncorrelated (ρ=0.00059), allowing us to independently evaluate their mediation pathways through CT imaging.

The first mediation analysis investigates the mediating role of CT imaging in the relationship between PRS and FEV1, achieving three aims: (A1) testing whether CT imaging mediates the effect of PRS on FEV1 (global mediation test), (A2) estimating the proportion of the TE mediated by CT imaging (global mediation percentage, GM%), and (A3) identifying and prioritizing specific lung regions contributing to the mediation effect. The global mediation test reveals a highly significant result ($p<10^{-16}$) and low neutralization rate (21%), confirming that CT imaging strongly mediates the effect of PRS on FEV1. The GM% is estimated to be 49%, indicating that nearly half of the TE of PRS on FEV1 is mediated by CT imaging. These results underscore the potential importance of lung imaging as a biomarker for genetic susceptibility to COPD. To further refine these insights, we identify 13 significant patches as active mediators using the Benjamini–Yekutieli procedure (q<0.01). Table 3, available as supplementary data at *Bioinformatics* online, lists the patch IDs, *q*-values, mediation contributions, and contribution proportions of the 13 significant patches.

The second mediation analysis evaluates PY as the environmental exposure, with the same three objectives. The global mediation test again shows a highly significant result ($p<10^{-16}$), confirming the strong mediating role of CT imaging in the relationship between smoking and FEV1. The neutralization rate was 18%, slightly lower than in the PRS analysis, indicating even greater consistency in the mediation contributions among lung patches. The GM% for PY is estimated to be 76%, considerably higher than the 49% observed in the PRS model. This result highlights the more substantial mediating role of CT imaging in the environmental pathway of smoking-related lung damage. We also identify 20 significant patches (q<0.01), listed in Table 3, available as supplementary data at *Bioinformatics* online.

**Figure 4.**
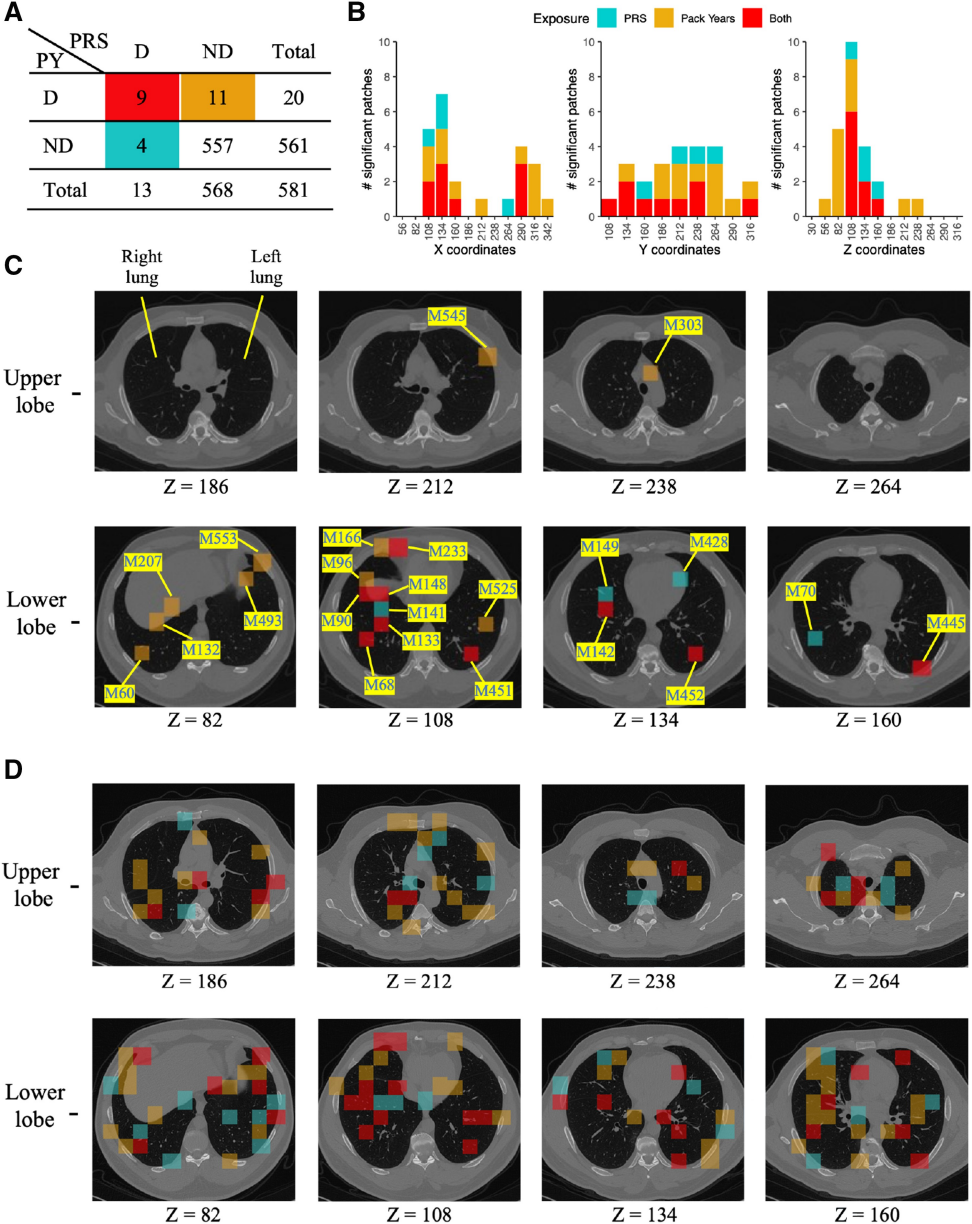
Visualization of COPD mediation analysis. (A) 2 × 2 contingency table of detected mediators from PRS-induced and PY-induced mediation analysis (D: detected, ND: non-detected). (B) Histogram of significant patches: the histogram displays the distribution of significant patches along the *X-*, *Y-*, and *Z*-coordinates of the lung image. (C) CT images on different *Z*-coordinates: These images visualize the most significant patches located in the lower lobe. (D) HIMA result on CT image: significant patches are scattered without regional clustering.

Given the independence of PRS and PY, we examine whether the mediation analyses identify overlapping lung regions. Among the 13 significant patches from the PRS analysis and the 20 significant patches from the PY analysis, nine were shared, suggesting common pathways through which genetic and environmental factors impact lung structure and function. A meta-analysis using Fisher’s method prioritizes the 25 unique mediators, and overlap enrichment is assessed using Fisher’s exact test. The results reveal a highly significant overlap (*p*-value $=5.7\times 10^{-12}$) with an odds ratio of 108.26, indicating strong enrichment of shared mediating regions. To visualize the spatial distribution of mediators, Fig. 4B shows histograms of the significant patches along the *X*, *Y*, and *Z* axes. The *Z*-coordinate shows that active mediators are predominantly located in the lower lung lobes (Z=56∼160). Figure 4C further illustrates the spatial clustering of active mediators, with four overlapping patches (M90, M148, M133, and M68) concentrated in the lower lobes at Z=108. Additionally, many significant patches across three nearby *Z*-coordinates (Z=82,108,134) share the same *X*- and *Y*-coordinates, providing strong evidence of stable mediator selection. For example, the patch pair (M132, M133), (M141, M142), and (M148, M149) are located at different *Z*-coordinates but occupy neighboring positions in the lung image, sharing identical *X*- and *Y*-coordinates. These findings suggest that specific subregions in the lower lung lobes play a critical role in mediating both genetic and environmental effects on COPD severity. Some patches (e.g. M233 and M428) lie outside the lung region due to visualization using a single subject. Our feature extraction method selects patches based on the average frequency of the patch that lies inside the lung across the population.

Our findings identify the lower lung lobes as significant CT imaging regions with strong mediation effects for both smoking and PRS exposures. While previous studies focus on upper lung regions due to their proximity to smoke inhalation (Takahashi *et al.* 2008), our findings suggest that the lower lobes may involve alternative mechanisms such as inflammation or immune responses and offer potential targets for therapies or interventions. This paradigm shift in understanding the role of CT imaging in COPD highlights the potential for advanced imaging techniques to inform disease progression, predict outcomes, and guide targeted treatments.

As a comparison, we also applied three popular methods (H&P, HILMA, and HIMA) to the COPDGene study. All of them detected a significant global indirect effect (*p*-value $<10^{-5}$), consistent with our findings. However, the significant patches identified by HIMA were scattered across the lung without highlighting any specific regions (e.g. lower or upper lobes), as shown in the 3D lung image in Fig. 4D. In contrast, our method not only detects the global mediation signal but also identifies spatially coherent clusters of active mediators in the lower lobe. By focusing on biologically meaningful and spatially localized mediators, our method provides results that are both statistically robust and clinically actionable.

## 5 Discussion

Causal mediation analysis is an essential tool in observational studies, allowing researchers to uncover the pathways through which an exposure affects an outcome via mediators. The emergence of high-dimensional data from omics and imaging studies creates a pressing need for frameworks that can handle these complex datasets, particularly the high correlations often observed among mediators. Our proposed PS5 framework addresses this need by answering three critical questions in high-dimensional causal mediation: (i) whether a global indirect effect is statistically significant, (ii) the proportion of the exposure–outcome association mediated by the set of candidate mediators, and (iii) the identification and ranking of active mediators based on their contributions. PS5 incorporates innovative statistical methodologies, including PS statistics and multiple sample splitting, to overcome challenges such as maintaining statistical power under varying mediation signal structures, detecting highly correlated true mediators, preserving causal assumptions, and accurately estimating mediation contributions. Through extensive simulations and an application to COPDGene imaging genetics data, PS5 demonstrates superior performance and reveals biologically meaningful insights compared to existing methods.

One important parameter in PS5 is γ in Equation (5), which influences the statistical power for detecting frequent or sparse signals. Simulations (see Supplementary Materials 4, available as supplementary data at *Bioinformatics* online) show that γ=2 (the setting used in this article) and γ=3 provide an optimal tradeoff, offering higher power for sparse and non-sparse signals compared to γ=1 and γ=4, respectively. This behavior aligns with findings in statistical frameworks employing heavy-tailed distribution transformations (Fang *et al.* 2024). While an adaptive bootstrap procedure described in He *et al.* (2024) could address conservative behavior under complete nulls, its computational costs would be substantial. Thus, we opted not to incorporate this approach into the current implementation.

Despite using sample splitting and Monte Carlo procedures, the computational efficiency of PS5 remains practical. For example, in the COPDGene application with *N* = 8897 patients and *P* = 5810 candidate mediators, the analysis of 500 multiple sample splits requires just over 9 min on a Dell server with 32 cores (Intel Xeon Gold 5218). This performance is competitive with existing methods and highlights the scalability of PS5, especially given its compatibility with parallel and GPU-based computing. Overall, PS5 offers a flexible foundation for high-dimensional causal mediation. Future extensions could incorporate spatial structures or correlations among mediators, particularly in imaging applications.

## Supplementary Material

btaf493_Supplementary_Data

## Data Availability

Chronic Obstructive Pulmonary Disease Genetic Epidemiology (COPDGene) data are available for request via COPD Gene Study. The proposed PS5 framework is developed into an R package available at: https://github.com/hung-ching-chang/PS5Med.
