# Supplementary material for: High-dimensional causal mediation analysis by partial sum statistic and sample splitting strategy in imaging genetics application
Source: Bioinformatics. 2025 Sep 10;41(10):btaf493. doi: 10.1093/bioinformatics/btaf493 (PMC12502916; doi:10.1093/bioinformatics/btaf493)
Supplement: btaf493_Supplementary_Data [file btaf493_supplementary_data.pdf]

Supplementary Materials for "High-Dimensional Causal Mediation Analysis by Partial Sum Statistic and Sample Splitting Strategy in Imaging Genetics Application"

Hung-Ching Chang, Yusi Fang, Michael T. Gorczyca, Kayhan Batmanghelich, George C. Tseng

S1 Proof of Proposition 1

Unlike ideally randomized experiments, which provide a direct way to infer causality, observational studies face greater challenges in establishing causal interpretations due to the absence of randomized treatment assignment (Hernán and Robins, 2020). Researchers rely on identification assumptions to establish causal effects (Pearl, 2001). VanderWeele and Vansteelandt (2014) propose four identification assumptions for multiple mediators. The sufficient assumptions for identifying causal effects in mediation analysis are: **(I)** $Y(x) \perp\!\!\!\perp X|\mathbf{C}$, **(II)** $Y(x, \mathbf{m}) \perp\!\!\!\perp \mathbf{M}|X, \mathbf{C}$, **(III)** $\mathbf{M}(x) \perp\!\!\!\perp X|\mathbf{C}$, and **(IV)** $Y(x, \mathbf{m}) \perp\!\!\!\perp \mathbf{M}(x^*)|\mathbf{C}$. Assumptions **(I)** – **(III)** are the no-unmeasured confounding assumptions, while assumption **(IV)** is known as the cross-world assumption. Notably, if any X-induced confounder is present, assumption (IV) may be violated even if it is observed (Andrews and Didelez, 2020). In reality, high-dimensional mediators, especially in omics and imaging data, often interact. Consequently, excluding partial mediators from the system can potentially lead to a violation of assumption **(IV)**. Thus, we should be cautious about dropping mediators from the joint system when reducing the dimensionality of mediators. The proof of Proposition 1 below demonstrates why our dimension reduction strategy preserves these causal assumptions, ensuring that mediation analysis remains valid after reducing the dimensionality of the mediators.

Proof. Denote X as exposure, Y as outcome, and $(M^{(1)}, ..., M^{(p)})$ as a p-dimensional mediator. Given the casual assumptions (I)-(IV) hold for multiple mediators model $X-$ $(M^{(1)}, ..., M^{(p)})$ $-Y$, we consider the corresponding Pearl's non-parametric structural equation model (Pearl, 2001) with a p-dimensional mediator:

$$X = f_X(\epsilon_X)$$
$$\mathbf{M} = f_{\mathbf{M}}(X, \epsilon_{\mathbf{M}})$$
$$Y = f_Y(X, \mathbf{M}, \epsilon_Y),$$

where $\mathbf{M} = (M^{(1)}, ..., M^{(p)})^T$, and the ϵ are noise terms. Here, for simplicity, we ignore covariates $\mathbf{C}$, but the proof can be extended with a suitable set of $\mathbf{C}$. With general setting of mediation model, noise terms are jointly independent $(\epsilon_X \perp\!\!\!\perp \epsilon_{\mathbf{M}}, \epsilon_X \perp\!\!\!\perp \epsilon_Y, \epsilon_{\mathbf{M}} \perp\!\!\!\perp \epsilon_Y)$ (Peters et al., 2017). Suppose that the $M^{(j)}$ is not the cause of the outcome, which means $M^{(j)}-Y$ relationship does not exist, i.e.,

$$Y = f_Y(X, \mathbf{M}^{(-j)}, \epsilon_Y).$$

We demonstrate the proof by considering $\mathbf{M}^{(-j)} = \{\mathbf{M}_{in}^{(-j)}, \mathbf{M}_{out}^{(-j)}, \mathbf{M}_{no}^{(-j)}\}$, where $\mathbf{M}_{in}^{(-j)}$, $\mathbf{M}_{out}^{(-j)}$,

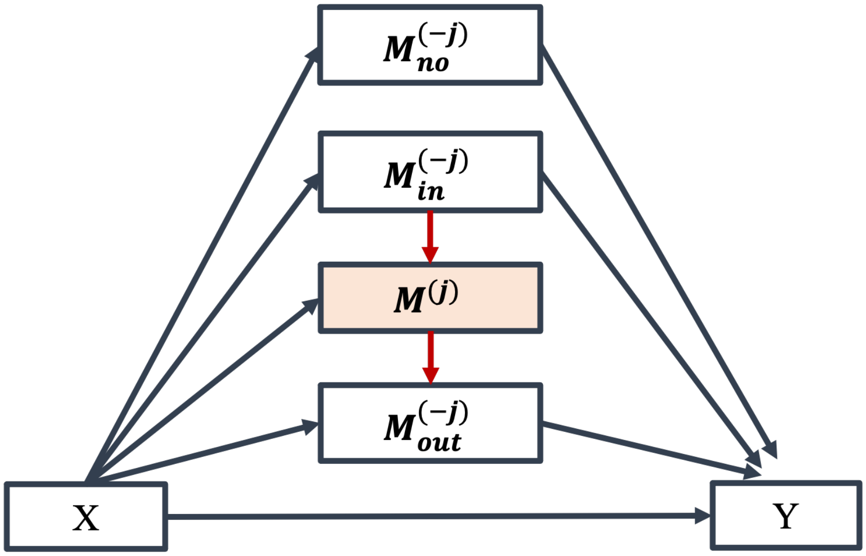

Figure S1: Example of a directed acyclic graph representing mediation effect through a set of mediators $\mathbf{M}$, but there is a candidate mediator $M^{(j)}$ without a mediator-outcome relationship.

and $\mathbf{M}_{no}^{(-j)}$ are the three distinct subsets: $\mathbf{M}_{in}^{(-j)}$ is the cause of $M^{(j)}$, $\mathbf{M}_{out}^{(-j)}$ is affected by $M^{(j)}$, and $\mathbf{M}_{no}^{(-j)}$ does not have direct interaction with $M^{(j)}$ (Figure S1). More general, $\mathbf{M}_{in}^{(-j)}$, $\mathbf{M}_{out}^{(-j)}$, and $\mathbf{M}_{no}^{(-j)}$ may interact with each other. Based on these, the model $\mathbf{M}$ can be divided into $M^{(j)}$ model, $\mathbf{M}_{in}^{(-j)}$ model, $\mathbf{M}_{out}^{(-j)}$ model, and $\mathbf{M}_{no}^{(-j)}$ model, i.e.,

$$\begin{aligned}
M^{(j)} &= f_{M^{(j)}}(X, \mathbf{M}_{in}^{(-j)}, \epsilon_{M^{(j)}}) \\
\mathbf{M}_{in}^{(-j)} &= f_{\mathbf{M}_{in}^{(-j)}}(X, \mathbf{M}_{out}^{(-j)}, \mathbf{M}_{no}^{(-j)}, \epsilon_{\mathbf{M}_{in}^{(-j)}}) \\
\mathbf{M}_{out}^{(-j)} &= f_{\mathbf{M}_{out}^{(-j)}}(X, M^{(j)}, \mathbf{M}_{in}^{(-j)}, \mathbf{M}_{no}^{(-j)}, \epsilon_{\mathbf{M}_{out}^{(-j)}}) \\
\mathbf{M}_{no}^{(-j)} &= f_{\mathbf{M}_{no}^{(-j)}}(X, \mathbf{M}_{in}^{(-j)}, \mathbf{M}_{out}^{(-j)}, \epsilon_{\mathbf{M}_{no}^{(-j)}}).
\end{aligned}$$

To simplify the result, we combine three $\mathbf{M}^{(-j)}$ models with considering sufficient input X, $M^{(j)}$, and $\epsilon_{\mathbf{M}^{(-j)}}$,

$$\mathbf{M}^{(-j)} = f_{\mathbf{M}^{(-j)}}(X, M^{(j)}, \epsilon_{\mathbf{M}^{(-j)}}),$$

where $\epsilon_{M^{(j)}} \perp\!\!\!\perp \epsilon_{\mathbf{M}^{(-j)}}$, $\epsilon_{M^{(j)}} \perp\!\!\!\perp \epsilon_X$, $\epsilon_{M^{(j)}} \perp\!\!\!\perp \epsilon_Y$, $\epsilon_{\mathbf{M}^{(-j)}} \perp\!\!\!\perp \epsilon_X$, and $\epsilon_{\mathbf{M}^{(-j)}} \perp\!\!\!\perp \epsilon_Y$. This system of equations implies the following potential outcome model:

$$\begin{aligned}
M^{(j)}(x) &= f_{M^{(j)}}(x, \mathbf{M}_{in}^{(-j)}(x), \epsilon_{M^{(j)}}) \\
oM^{(j)}(x^*) &= f_{M^{(j)}}(x^*, \mathbf{M}_{in}^{(-j)}(x^*), \epsilon_{M^{(j)}}) \\
\mathbf{M}^{(-j)}(x^*) &= f_{\mathbf{M}^{(-j)}}(x^*, M^{(j)}(x^*), \epsilon_{\mathbf{M}^{(-j)}}) \\
Y(x, \mathbf{m}^{(-j)}) &= f_Y(x, \mathbf{m}^{(-j)}, \epsilon_Y)
\end{aligned}$$

This implies that assumption (IV) hold, $Y(x, \mathbf{m}^{(-j)}) \perp\!\!\!\perp \mathbf{M}^{(-j)}(x^*)$, since they do not share the same structure of the joint distribution.

In summary, if $M^{(j)}$ is not a parent of Y, then it appears trivial that the four assumptions hold under Rubin's potential outcomes framework. Particularly if these four assumptions are made prior to removal of $M^{(j)}$. Assumption (I) doesn't concern the mediator-outcome relationship. Assumption (II) should hold by consistency, as $M^{(j)}$ would not become confounding variables for

the mediator-outcome relationship. Assumption (III) has nothing to do with the mediator-outcome relationship. Assumption (IV) should again hold by consistency. □

S2 Biological interpretation of neutralization ratio (NR)

Conceptually, a high NR indicates strong cancellation, where mediators act in opposing directions, while a low NR reflects minimal cancellation, with most mediators acting in the same direction. Biologically, high NR values may point to competing processes across mediators. For example, some regions contribute to tissue damage while others counteract it. In contrast, a low NR suggests a more consistent process among mediators in the system that either worsens or improves disease severity. Supplementary Table S1 gives four simple examples to illustrate how the NR reflects the balance between positive and negative indirect effects. In Example 1 (NR = 100%), the positive and negative effects are exactly equal, giving complete cancellation. Example 2 (NR = 66.7%) shows partial cancellation, with one direction dominating. Example 3 (NR = 33.3%) has only limited cancellation, with most mediators acting in the same direction. Example 4 (NR = 0%) shows no cancellation at all, with all mediators producing the same direction of effect.

	Mediation Contribution ($\alpha\beta$)			
Mediators	Example 1	Example 2	Example 3	Example 4
M_1	1	1	1	1
M_2	2	2	2	2
M_3	-1	1	-1	1
M_4	-2	-2	2	-2
$\lvert IE^{+}\rvert$	3	4	5	6
$\lvert IE^{-}\rvert$	3	2	1	0
IE	0	2	4	6
NR	100%	66.7%	33.3%	0%

Table S1: Four examples showing how the neutralization ratio (NR) ranges from complete cancellation (NR = 100%) to no cancellation (NR = 0%).

S3 Table of type I error rate

We set the significance level at p-value < 0.05. Table S2 shows that only PS5 can control type I error under 5% across all scenarios. For complete nulls, only HILMA is anti-conservative, while the other three methods are overly conservative. HIMA and HILMA are severely anti-conservative under dense nulls due to the problem of over-optimism. The only method in category II, the H&P method, shows a conservative result under sparse nulls. Lastly, our method is the only one that can control type I error under disjunctive nulls.

	Null 1 Complete nulls		Null 2 Dense nulls		Null 3 Sparse nulls		Null 4 Disjunctive nulls	
	$\rho = 0$	$\rho = 0.5$	$\rho = 0$	$\rho = 0.5$	$\rho = 0$	$\rho = 0.5$	$\rho = 0$	$\rho = 0.5$
PS5	0.00%†	0.00%†	4.50%	5.45%	3.10%	3.75%	4.85%	5.65%
H&P	0.00%†	0.00%†	5.15%	4.47%	0.00%†	0.00%†	75.05%‡	100%‡
HIMA	1.90%†	1.80%†	99.80%‡	99.65%‡	4.75%	4.35%	29.65%‡	18.85%‡
HILMA	36.00%‡	40.00%‡	100%‡	100%‡	3.00%	5.00%	10.00%‡	6.00%‡

†: conservative

‡: inflated

Table S2: Type I error results for continuous exposure under four null cases and two correlation settings ($\rho = 0, 0.5$).

S4 Comparison of γ parameter

We consider four γ parameters ($\gamma = 1, 2, 3, 4$), which represent the $L1$, $L2$, $L3$, and $L4$ norm of $\alpha_{Xj}\beta_{Mj}$ in null hypothesis and partial sum statistic, under non-correlation setting ($\rho = 0$) and four signal structures ($|\mathcal{S}|/p = 0.5\%$, 1%, 3%, and 5%). Figure S2 shows that $\gamma = 2, 3, 4$ provides roughly 10% higher power than $\gamma = 1$ for detecting sparse signal ($|\mathcal{S}|/p = 0.5\%$ and 1%). A larger γ increases the influence of the one or several strongest signals and is thus more powerful for sparse signal. On the other hands, the power of $\gamma = 1, 2, 3$ are more powerful than $\gamma = 4$ for detecting non-sparse signal ($|\mathcal{S}|/p = 5\%$). Thus, $\gamma = 2$ and $\gamma = 3$ provide an optimal trade-off for detecting both sparse and non-sparse signals.

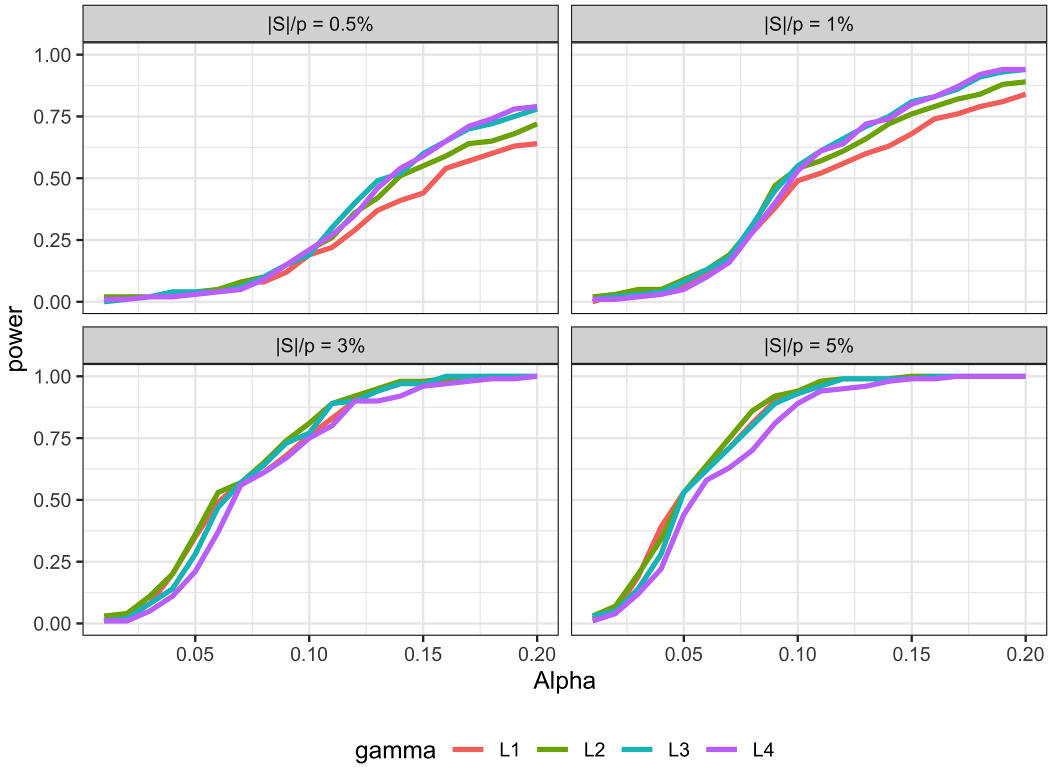

Figure S2: Power of two γ settings for detecting global indirect effect under four signal structures ($|\mathcal{S}|/p = 0.5\%$, 1%, 3%, and 5%).

S5 Table of top mediators for PRS and Pack Years exposures in COPDGene study

Supplement Table S3 lists the patch IDs, BH adjusted p-value (q-values), mediation contributions, and contribution proportions of the 25 significant patches identified by PRS and/or Pack Years analysis.

	q-value		Mediation Contribution		Contribution Proportion		Fisher's Combined
Patch index	PRS	Pack Years	PRS	Pack Years	PRS	Pack Years	q-value
M90-1	**2.49e-04****	**2.24e-04****	-0.2856	-0.1728	4.94%	4.78%	**9.90e-07****
M142-1	**2.79e-04****	**2.02e-04****	-0.1504	-0.1216	2.60%	3.38%	**1.00e-06****
M233-1	**3.43e-04****	**2.59e-04****	-0.1361	-0.1120	2.35%	3.10%	**1.53e-06****
M451-1	**3.44e-04****	**6.82e-04****	-0.2423	-0.1280	4.19%	3.52%	**3.82e-06****
M133-3	**2.12e-03***	**2.58e-04****	-0.1156	-0.1536	2.00%	4.25%	**8.46e-06****
M452-1	**1.86e-03***	**1.33e-03***	-0.2332	-0.1104	4.03%	3.08%	**3.47e-05****
M68-1	**4.36e-03***	**1.76e-03***	-0.1640	-0.1184	2.83%	3.27%	**9.85e-05****
M148-1	**2.16e-03***	**3.64e-03***	-0.2188	-0.2112	3.78%	5.81%	**1.00e-04****
M428-1	**2.08e-03***	1.24e-02	-0.1230	-0.0592	2.12%	1.64%	**3.01e-04****
M445-3	**6.63e-03***	**4.77e-03***	-0.1040	-0.0688	1.79%	1.91%	**3.59e-04****
M60-2	1.41e-02	**6.95e-03***	-0.0912	-0.0624	1.57%	1.73%	**1.00e-03***
M133-1	4.78e-02	**2.25e-03***	-0.0915	-0.1024	1.58%	2.83%	**1.09e-03***
M525-2	1.39e-02	**8.58e-03***	-0.0978	-0.0784	1.69%	2.16%	**1.20e-03***
M149-2	**9.73e-03***	1.45e-02	-0.1945	-0.0928	3.36%	2.56%	**1.39e-03***
M166-2	4.96e-02	**8.53e-03***	-0.0727	-0.0640	1.25%	2.04%	**3.71e-03***
M493-8	1.18e-01	**4.32e-03***	-0.0286	-0.0464	0.49%	1.29%	**4.39e-03***
M545-1	1.00e-00	**7.56e-04****	-0.0133	-0.1328	0.23%	3.67%	**6.19e-03***
M70-1	**4.45e-03***	2.20e-01	-0.1880	-0.0864	3.25%	2.37%	**7.78e-03***
M132-4	1.93e-01	**5.83e-03***	-0.0361	0.0528	0.62%	1.46%	**8.79e-03***
M579-1	8.57e-01	**2.01e-03***	0.0396	-0.0896	0.68%	2.48%	1.26e-02
M207-1	6.30e-01	**3.02e-03***	-0.0744	-0.1600	1.28%	4.42%	1.38e-02
M303-1	1.00e-00	**2.57e-03***	-0.0198	-0.0896	0.34%	2.47%	1.79e-02
M141-2	**2.81e-03***	1.00e-00	-0.1346	-0.0160	2.33%	0.48%	1.93e-02
M553-2	1.00e-00	**3.29e-03***	-0.0306	-0.1072	0.52%	2.98%	2.21e-02
M96-2	1.00e-00	**5.57e-03***	-0.0146	-0.0864	0.25%	2.40%	3.44e-02

Note: "*" denotes q-value < 0.01; "**" denotes q-value < 0.001.

Table S3: Top mediators for PRS and Pack Years exposures ordered by Fisher's Combined q-value.

S6 Detailed algorithm flowchart

To address the statistical challenges and achieve the three aims (A1–A3), we propose a novel framework (PS5) for estimating and testing the global mediation effect, as well as prioritizing mediators based on their p-values. Supplementary Figure S3 presents a step-by-step flowchart of the PS5 procedure, summarizing each stage described in the Methods Section.

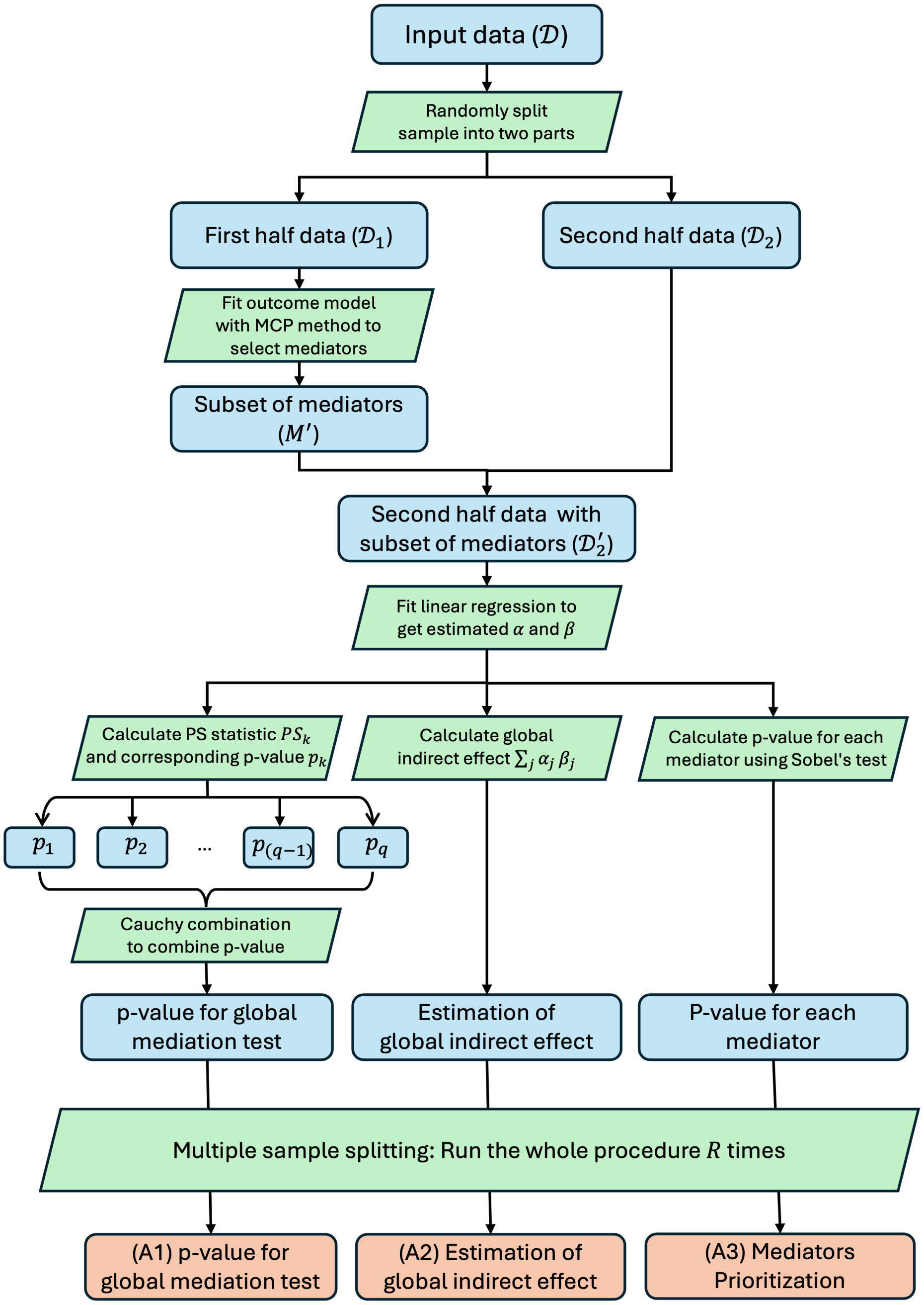

Figure S3: Flowchart of PS5. First, we perform sample splitting and select mediators using MCP methods. Second, we fit linear regression to estimate mediation effect. Third, we calculate PS statistic to test global indirect effect.

S7 Additional sensitivity results for $|\mathcal{S}|/p = 1\%$ and 3%

We provide two additional sensitivity results for different signal structures ($|\mathcal{S}|/p = 1\%$ and 3%) in Figure S4. Regardless of signal structures, PS5 can reach 100% sensitivity as $\alpha_{\mathbf{X}}$ magnitude increases. However, HIMA cannot select all true mediators even if the signal strength is strong.

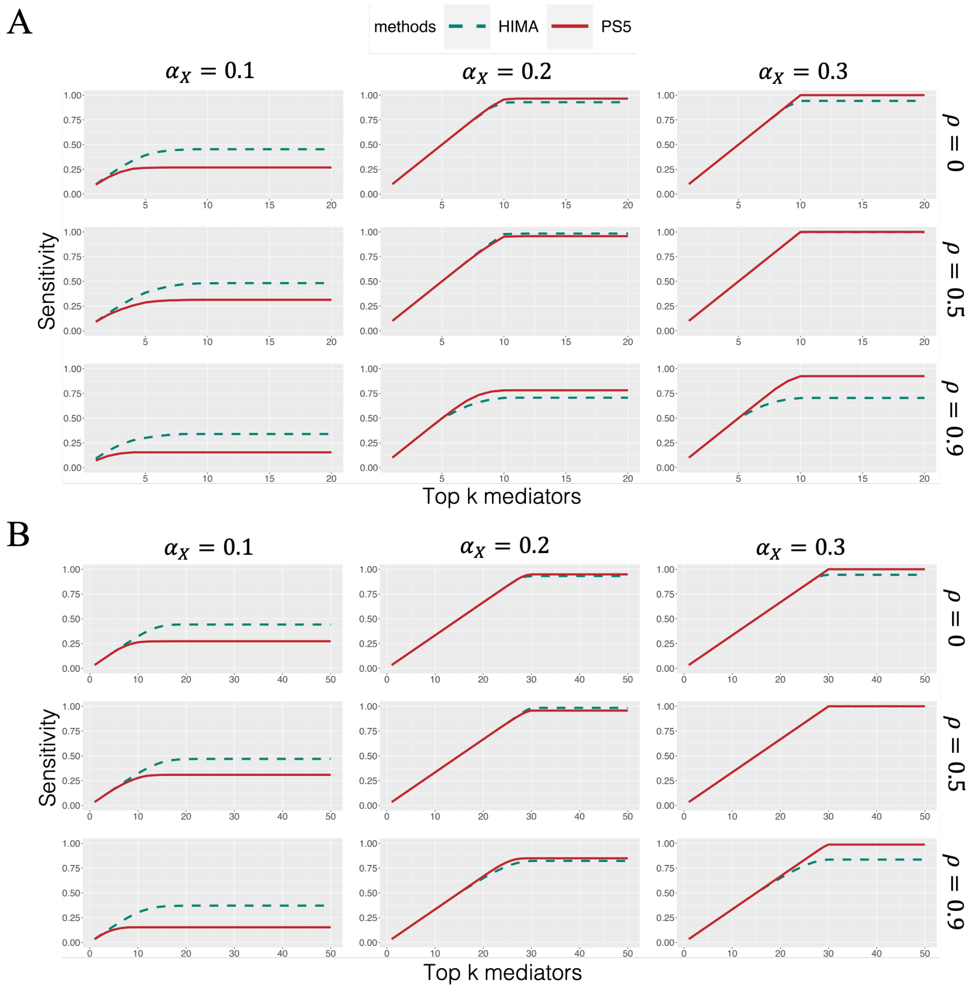

Figure S4: Accuracy of mediator prioritization for continuous exposure by sensitivity. (A) $|\mathcal{S}|/p = 1\%$ (B) $|\mathcal{S}|/p = 3\%$

S8 Estimation bias of global indirect effect with an unmeasured confounder U

To assess possible violations due to confounding variables, we performed an additional sensitivity analysis under the continuous exposure setting, similar to that used in Clark-Boucher et al. (2023).

We considered an unmeasured confounding variable U that is generated from $N(0, \tau)$ and impacts exposure X, mediator $\mathbf{M}$, and outcome Y in the data-generating procedure:

$$X_i = \delta U_i + \epsilon_{X_i}$$
$$\mathbf{M}_i = \alpha_{\mathbf{C}} \mathbf{C}_i + X_i \alpha_{\mathbf{X}} + U_i \alpha_{\mathbf{U}} + \epsilon_{\mathbf{M}_i}$$
$$Y_i = \mathbf{C}_i^T \beta_{\mathbf{C}} + X_i \beta_X + \mathbf{M}_i^T \beta_{\mathbf{M}} + U_i \beta_{\mathbf{U}} + \epsilon_{Y_i},$$

where $\delta = 0.5$, $\alpha_{\mathbf{U}} = \alpha_{\mathbf{X}}/2$, and $\beta_{\mathbf{U}} = \beta_{\mathbf{M}}/2$ if $\beta_M \neq 0$. According to Figure 3B in the main manuscript, the estimation bias does not change much as long as $\alpha_X > 0.1$, so we fix α_X to be 0.1. H&P method is no longer considered in this comparison since it has a significantly larger bias than the others. Figure S5 illustrates that all methods show a larger bias in global indirect effect estimation as the variance of the unmeasured confounding variable (τ) increases. Among the three methods, HILMA is the most robust, but our proposed method is also comparable to HILMA as the number of true mediators $|S|$ increases.

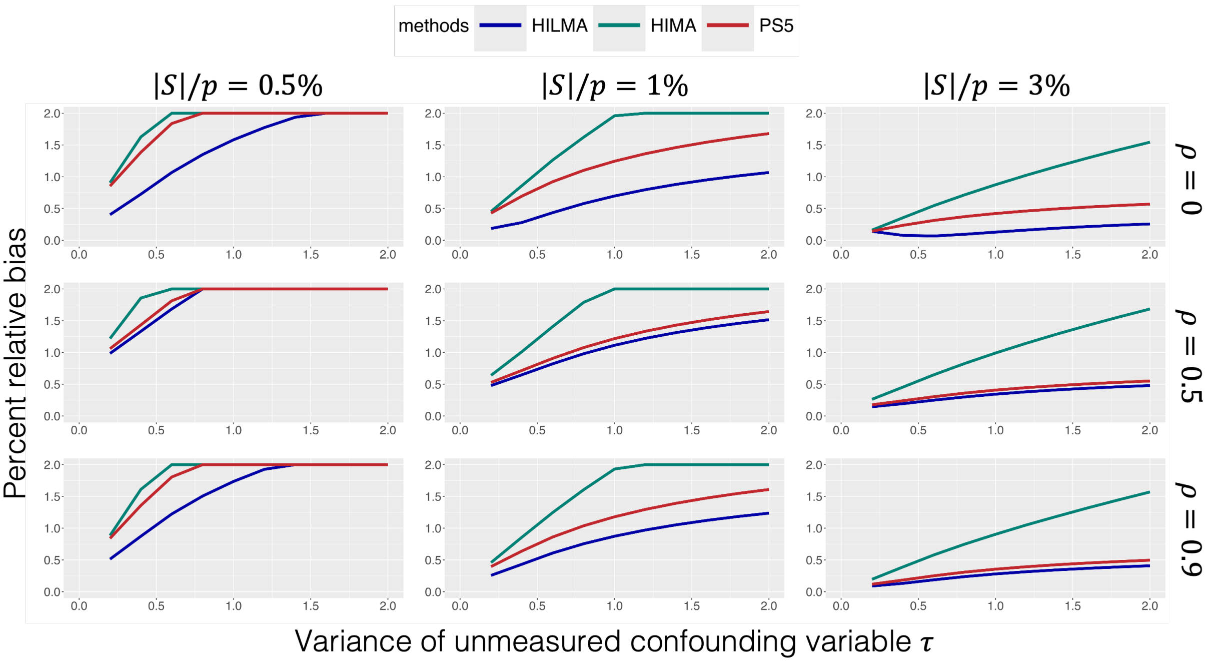

Figure S5: Percent relative bias for estimating global indirect effect when unmeasured confounding variable exists. The y-axis is truncated at 200%.

S9 Simulation results for discrete exposure

To mimic the SNPs exposure, we also do the comprehensive simulation by randomly sampling discrete exposure X from 0, 1, and 2. All other parameter settings remained consistent with those in the main article. We present results for type I error, power, relative bias, and accuracy of mediator prioritization in Table S4, Figure S6, and Figure S7.

For type I error control (Table S4), only PS5 successfully controls type I error under 5% across all scenarios, while other methods tend to be inflated or conservative. Under Null 1, all methods have conservative type I error. For the power of global mediation test (Figure S6A), HIMA and PS5 have higher power than HILMA and H&P. However, as the number of true signals $|\mathcal{S}|$ increases, HILMA and PS5 become the most powerful methods across all four methods. Overall, PS5 was the only method capable of effectively detecting both sparse and non-sparse signals.

	Null 1 Complete nulls		Null 2 Dense nulls		Null 3 Sparse nulls		Null 4 Disjunctive nulls	
	$\rho = 0$	$\rho = 0.5$	$\rho = 0$	$\rho = 0.5$	$\rho = 0$	$\rho = 0.5$	$\rho = 0$	$\rho = 0.5$
PS5	0.00%†	0.00%†	5.60%	5.90%	3.25%	3.95%	5.60%	5.30%
H&P	0.00%†	0.00%†	5.10%	5.10%	0.00%†	1.20%†	100%‡	100%‡
HIMA	1.80%†	1.55%†	82.1%‡	80.6%‡	4.35%	4.25%	10.30%‡	10.90%‡
HILMA	61.05%‡	65.15%‡	100%‡	100%‡	1.95%†	4.70%	4.00%	4.00%

†: conservative

‡: inflated

Table S4: Type I error results for discrete exposure. We consider four scenarios and two correlation settings ($\rho = 0, 0.5$) under the null.

For relative estimation bias of the global mediation effect (Figure S6B), PS5 would be the best-performing method and roughly $10 \sim 20\%$ lower than HILMA and HIMA under non-correlation settings. In the other settings with correlated mediators, both PS5 and HILMA can achieve lower estimation bias, which outperforms HIMA by approximately 10%. Among these three methods, the relative bias of H&P tends to be much higher, regardless of the correlation structures. For the accuracy of mediator prioritization, we present the result by sensitivity, and Figure S7 shows that only PS5 can achieve 100% sensitivity when $\alpha_{\mathbf{X}}$ magnitude increases.

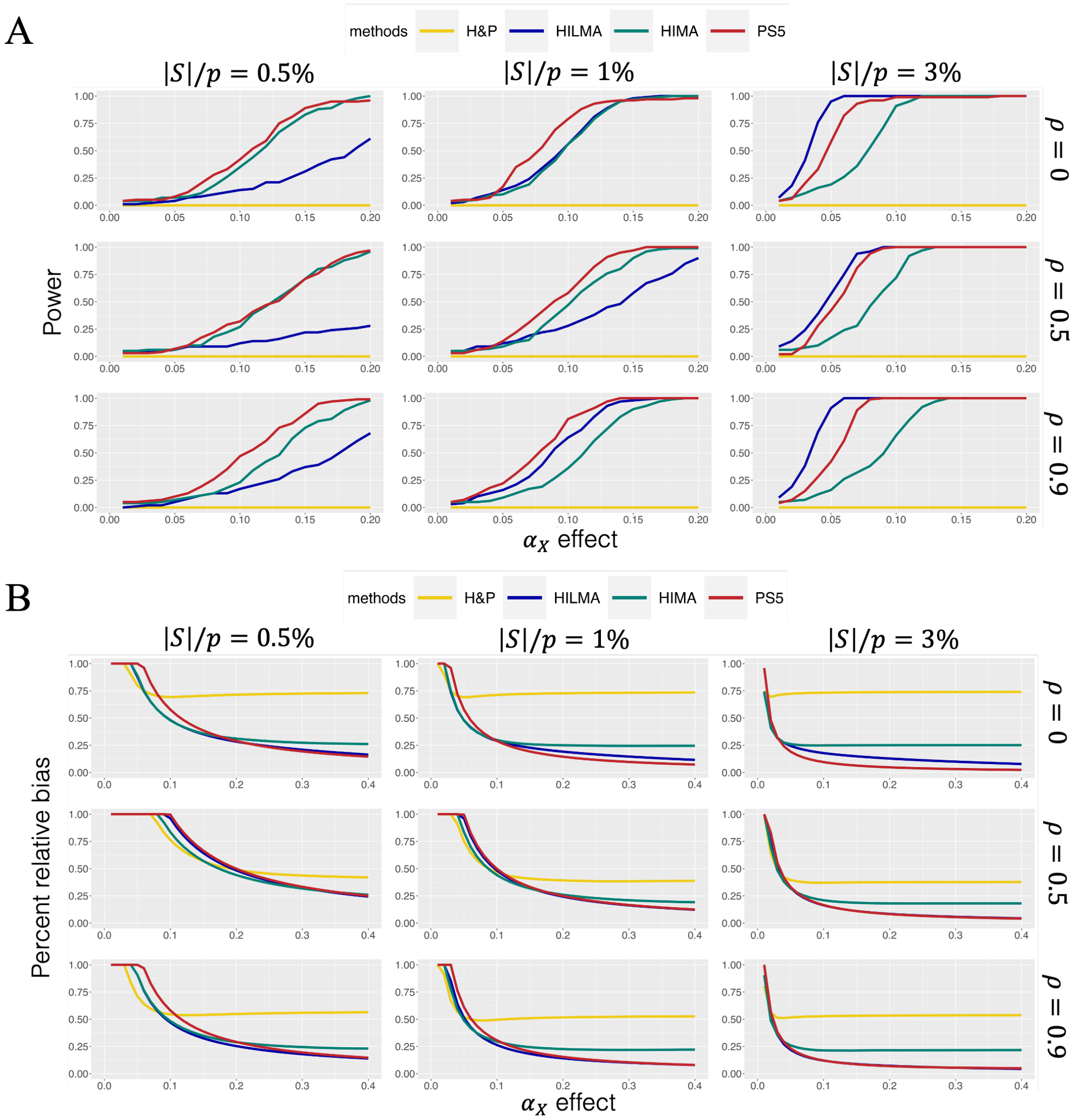

Figure S6: Simulation results for count exposure. (A) Power for detecting global indirect effect (B) Percent relative bias for estimating global indirect effect

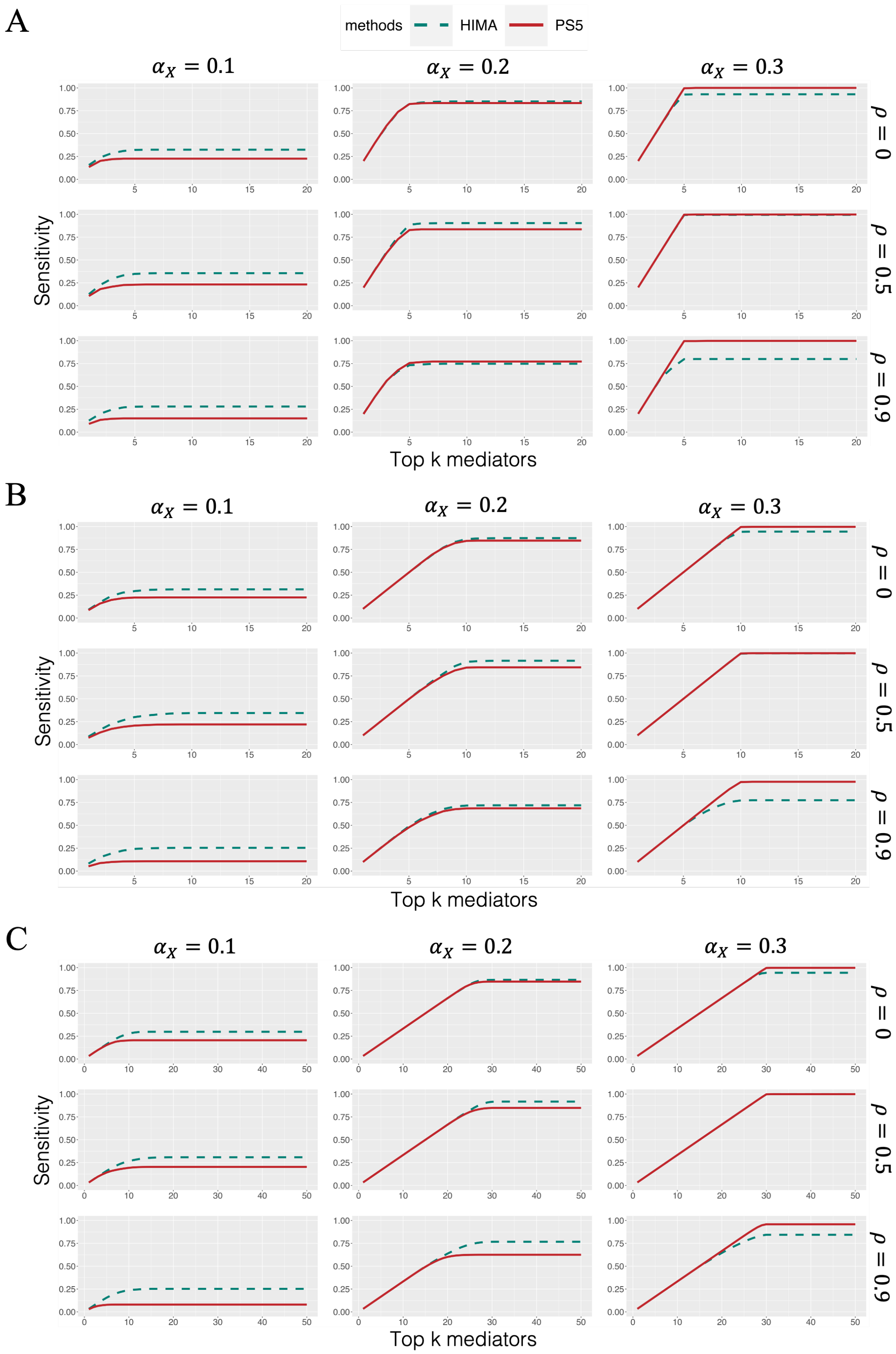

Figure S7: Accuracy of mediator prioritization for count exposure by sensitivity. (A) $|\mathcal{S}|/p = 0.5\%$ (B) $|\mathcal{S}|/p = 1\%$ (C) $|\mathcal{S}|/p = 3\%$

References

Andrews, R. M. and Didelez, V. (2020). Insights into the cross-world independence assumption of causal mediation analysis. *Epidemiology*, 32(2):209–219.

Clark-Boucher, D., Zhou, X., Du, J., Liu, Y., Needham, B. L., Smith, J. A., and Mukherjee, B. (2023). Methods for mediation analysis with high-dimensional dna methylation data: Possible choices and comparison. *medRxiv*, pages 2023–02.

Hernán, M. and Robins, J. (2020). Causal inference: What if. *Boca Raton: Chapman & Hall/CRC*.

Pearl, J. (2001). Direct and indirect effects. In *Proceedings of the Seventeenth Conference on Uncertainty and Artificial Intelligence*, pages 411–420.

Peters, J., Janzing, D., and Schölkopf, B. (2017). *Elements of causal inference: foundations and learning algorithms*. The MIT Press.

VanderWeele, T. and Vansteelandt, S. (2014). Mediation analysis with multiple mediators. *Epidemiologic Methods*, 2(1):95–115.
